# Supplementary material for: Reconciling Mining with the Conservation of Cave Biodiversity: A Quantitative Baseline to Help Establish Conservation Priorities
Source: PLoS One. 2016 Dec 20;11(12):e0168348. doi: 10.1371/journal.pone.0168348 (PMC5173368; doi:10.1371/journal.pone.0168348)
Supplement: S1 Dataset — (ZIP) [file pone.0168348.s002.zip › Taxa/Serra Norte/SN_2013/N4E/Lista N4E-68.pdf]

CAVIDADE N4E-0068

Classe	Ordem	Fam/Outros	Gên/Outros	Espécie	S-U	U-S	Zon.
Arachnida	Acari	Cunaxidae		sp.1	X		P
Arachnida	Acari	Mesostigmata		jovem	X		P
Arachnida	Acari	Veigaiidae		sp.1	X		P
Arachnida	Amblypygi	Charinidae	*Charinus*	sp.1	2		P
Arachnida	Amblypygi	Phrynidae	*Heterophrynus*	*longicornis*	2		P
Arachnida	Araneae	Caponiidae		jovem		X	A
Arachnida	Araneae	Ctenidae		jovem	3		P
Arachnida	Araneae	Pholcidae	*Mesabolivar*	*aurantiacus*	X		E
Arachnida	Araneae	Pholcidae	*Mesabolivar*	sp.1	X		E
Arachnida	Araneae	Pisauridae	*Thaumasia*	*velox*	4		P
Arachnida	Araneae	Theridiosomatidae	*Plato*	sp.1	X	X	E/P
Arachnida	Opiliones	Escadabiidae		sp.2	X	X	P/A
Arachnida	Opiliones	Sclerosomatidae	*Prionostema*	sp.1	3		E
Arachnida	Pseudoscorpiones	Bochicidae		sp.1	X	X	A
Diplopoda	Polydesmida	Fuhrmannodesmidae		jovem	X		P
Diplopoda	Polydesmida	Pyrgodesmidae		sp.1	X	X	P/A
Entognatha	Collembola	Isotomidae		sp.1		X	A
Entognatha	Collembola	Paronellidae		sp.4	X	X	P/A
Insecta	Coleoptera	Carabidae		sp.3	X		A
Insecta	Coleoptera	Dytiscidae		sp.17	X		A
Insecta	Coleoptera	Dytiscidae		sp.9	X		A
Insecta	Coleoptera	Hydrophilidae		sp.10	X		A
Insecta	Coleoptera	Staphylinidae	Pselaphinae	sp.12		X	A
Insecta	Diptera	Chironomidae		sp.	X	X	P
Insecta	Diptera	Phoridae	Metopininae	sp.	X		A
Insecta	Diptera	Phoridae	Phorinae	sp.		X	P/A
Insecta	Diptera	Tipulidae	Tipulinae	sp.	X		E
Insecta	Heteroptera	Gerridae	*Bachymetra*	jovem	X		P
Insecta	Heteroptera	Notonectidae		sp.	X		A
Insecta	Heteroptera	Reduviidae	Emesinae	sp.6	1		
Insecta	Heteroptera	Veliidae	*Paravelia*	sp.	X	X	P/A
Insecta	Heteroptera	Veliidae	*Rhagovelia*	sp.	X		P
Insecta	Hymenoptera	Diapriidae		sp.1	X		E/P
Insecta	Hymenoptera	Formicidae	*Paratrechina*	sp.2	X		E
Insecta	Hymenoptera	Formicidae	*Solenopsis*	sp.1	X		E
Insecta	Isoptera	Termitidae	*Nasutitermes*	sp.	X		E
Insecta	Lepidoptera			jovem	X		P/A
Insecta	Mantodea	Mantidae		sp.1	1		E
Insecta	Orthoptera	Gryllidae	Nemobiinae	sp.		3	
Insecta	Orthoptera	Phalangopsidae	*Phalangopsis*	sp.	54	70	E/P/A
Insecta	Thysanura	Nicoletiidae		sp.1		X	A
Gastropoda	Pulmonata	Subulinidae		sp.2	X		P
Symphyla		Scutigerellidae		sp.	X		P/A
Amphibia	Anura	Strabomantidae	*Pristimantis*	cf. *fenestratus*	4		E
Mammalia	Chiroptera	Furipteridae	*Furipterus*	*horrens*	10		P/A
Mammalia	Chiroptera	Phyllostomidae	*Carollia*	*perspicillata*	1	50	P/A
Mammalia	Chiroptera	Phyllostomidae	*Diphylla*	*ecaudata*	5		P/A
Mammalia	Rodentia	Cricetidae	*Rhipidomys*	sp.	1		P
